# Posttraumatische Belastungsstörung bei Kindern und Jugendlichen: Ergebnisse einer Querschnittsstudie zu Auswirkungen der neu formulierten Diagnosen PTBS und kPTBS in der ICD-11

**DOI:** 10.1007/s00103-024-03860-2

**Published:** 2024-03-18

**Authors:** Rebekka Eilers, Verena Ertl, Barbara Kasparik, Anne Kost, Rita Rosner

**Affiliations:** 1 Institut für Psychologie, Katholische Universität Eichstätt-Ingolstadt, Eichstätt, Deutschland; 2 Altonaer Kinderkrankenhaus, Kinder- und Jugendsomatik, Hamburg, Deutschland; 3 Katholische Universität Eichstätt-Ingolstadt, Ostenstr. 25, 85072 Eichstätt, Deutschland

**Keywords:** Posttraumatische Belastungsstörung, Komplexe posttraumatische Belastungsstörung, Kinder und Jugendliche, Diagnostische Kriterien, Entwicklungssensitive Diagnostik, Posttraumatic stress disorder, Complex posttraumatic stress disorder, Children and adolescents, Diagnostic criteria, Developmentally sensitive diagnostics

## Abstract

**Hintergrund:**

Die in der ICD-11 enger gefassten Kriterien der posttraumatischen Belastungsstörung (PTBS) und die Einführung der komplexen PTBS (kPTBS) mit zusätzlichen Schwierigkeiten in der Selbstorganisation und -regulation (SSO) können deutliche Auswirkungen auf die Diagnosehäufigkeit haben. In der vorliegenden Studie wurde untersucht, aufgrund welcher ICD-11-Cluster Kinder und Jugendliche die Diagnose verfehlen und ob Bezugspersonen Veränderungen im SSO-Bereich eher auf den Entwicklungsstand oder das traumatische Ereignis attribuieren und wie diese Attributionen wiederum mit der Symptomschwere zusammenhängen.

**Methoden:**

*N* = 88 deutschsprachige Kinder und Jugendliche (Alter: 7–17) mit traumatischen Ereignissen sowie *N* = 79 Bezugspersonen wurden zwischen September 2019 und November 2020 zur (k)PTBS-Symptomschwere (CATS-2) und der Attribution der SSO-Symptome (Fragebogen für Bezugspersonen) befragt.

**Ergebnisse:**

Die ICD-11-Kriterien (CATS‑2 und eine entwicklungsangepasste Version) ergaben geringere Häufigkeitsraten der PTBS als DSM‑5 und ICD-10. Am seltensten wurden die ICD-11-Cluster „Wiedererleben“ und „Übererregung“ erfüllt. Veränderungen der SSO-Symptome wurden vorwiegend als ereignisbedingt eingeschätzt. Diese Attribution hing mit höherer PTBS- und SSO-Symptomschwere im Fremdbericht zusammen. Die entwicklungsbedingte Attribution hing mit einer höheren SSO-, jedoch nicht PTBS-Symptomschwere im Fremdbericht zusammen.

**Diskussion:**

Im Rahmen der Diagnostik und bei der Überarbeitung von Diagnoseinstrumenten für ICD-11-(k)PTBS sollten auch entwicklungsspezifische Symptomausprägungen berücksichtigt werden. Eine Herausforderung stellt die Abgrenzung von Veränderungen im SSO-Bereich als „traumabezogen“ gegenüber „entwicklungsbedingt“ dar und erfordert mehrere Informationsquellen.

## Abstract (en)

**Background:**

ICD-11 presents narrowed criteria for posttraumatic stress disorder (PTSD) and introduces complex PTSD (CPTSD) with additional difficulties in self-organization (DSO). These changes can have significant effects on the frequency of the diagnosis. The aim of this study was to investigate which ICD-11 symptom clusters cause children and adolescents to miss the diagnosis and whether caregivers are more likely to attribute changes in DSO to developmental level or to the traumatic event, and how these attributions are in turn related to symptom severity.

**Methods:**

*N* = 88 German-speaking children and adolescents (age: 7–17 years) after traumatic events and *N* = 79 caregivers participated between September 2019 and November 2020 in a survey on PTSD symptom severity (CATS-2) and attribution of DSO symptoms (caregiver questionnaire).

**Results:**

The ICD-11 criteria (CATS‑2 and a developmentally adapted version) showed lower frequency rates for PTSD as compared to DSM‑5 and ICD-10. The ICD-11 clusters re-experiencing and hyperarousal were met the least often. Changes in DSO symptoms were predominantly rated as event-related. This attribution was associated with higher PTSD and DSO symptom severity in caregiver reports. The age-related attribution was associated with higher DSO-symptom severity, but not PTSD symptom severity in caregiver reports.

**Discussion:**

In the context of the diagnostic process and the revision of diagnostic instruments for ICD-11 (C)PTSD, development-specific symptoms should be taken into account. The trauma-related differentiation of DSO symptom changes as compared to development-related fluctuations is challenging and therefore requires several sources of information.

## Hintergrund

Mit der neuen 11. Auflage der „Internationalen statistischen Klassifikation der Krankheiten und verwandter Gesundheitsprobleme“ (ICD-11; [1]) wurden die Ziele einer verbesserten internationalen Anwendbarkeit, wissenschaftlichen Validität und klinischen Nützlichkeit der Diagnosekriterien verfolgt [2]. Die Kapitelstruktur wurde an die 5. Auflage des „Diagnostic and Statistical Manual of Mental Disorders“ (DSM‑5; [3]) angepasst [4, 5], dies führte zur deutlichen Umstrukturierung der Störungen des Kindes- und Jugendalters und zur Aufgabe des vormaligen Kapitels F9 „Verhaltens- und emotionale Störungen mit Beginn in der Kindheit und Jugend“. Einzelne der bislang hierunter aufgeführten Störungsbilder sind in anderen Kapiteln untergekommen. Beispielsweise fällt die Aufmerksamkeitsdefizit- und Hyperaktivitätsstörung nun unter die neuronalen Entwicklungsstörungen. Emotionale Störungen des Kindesalters sind teilweise im Kapitel der Angst- und furchtbezogenen Störungen zu finden, z. B. die Trennungsangst. Andere Störungsbilder des Kindes- und Jugendalters sind in der ICD-11 nicht mehr enthalten, wie die „Generalisierte Angststörung des Kindesalters“ oder die „Störung mit sozialer Ängstlichkeit des Kindesalters“.

Auch für die posttraumatische Belastungsstörung (PTBS, ICD-11 6B40) ergaben sich im Rahmen der ICD-11 deutliche Veränderungen. Diese waren teilweise eine Reaktion auf die Kritik an anderen Versionen der PTBS-Kriterien aufgrund einer breiten Zusammensetzung der Symptomcluster, hoher Komorbiditätsraten und einer hohen Anzahl möglicher Symptomkombinationen, die zur Diagnose führen konnten [6]. In der ICD-11 wurde die PTBS auf 6 Kernsymptome reduziert, die jeweils 2 Symptome in den 3 Clustern „Wiedererleben“, „Vermeidung“ und „Übererregung“ umfassen. Mit Einführung der „komplexen PTBS“ (kPTBS, ICD-11 6B41) als zusätzliche neue Diagnose wurden zudem weitere Symptome, die im Rahmen einer posttraumatischen Stressreaktion auftreten können und die auf Basis von Feldstudien, Expertenurteilen und einer Umfrage unter Praktiker:innen ermittelt wurden, berücksichtigt [6–11]. Gleichzeitig wurde die ICD-10-Diagnose der andauernden Persönlichkeitsänderung nach Extrembelastung aufgegeben, da diese Diagnose in der Forschung kaum Berücksichtigung fand [6]. Die kPTBS wird durch das Vollbild einer PTBS nach ICD-11 und zusätzliche Schwierigkeiten der Selbstorganisation und -regulation (SSO) beschrieben. SSO umfassen 6 Symptome in 3 Clustern: Probleme der Affektregulation, negatives Selbstbild und Schwierigkeiten der Beziehungsgestaltung [1, 6]. Zur Diagnosestellung der ICD-11-PTBS und auch -kPTBS muss mindestens ein Symptom pro Cluster ausgeprägt sein. Wenn die SSO-Symptome zusätzlich zur PTBS erfüllt sind, so wird nach ICD-11 die kPTBS kodiert. Damit unterscheidet sich die Konzeption der (k)PTBS-Diagnosen in ICD-11 deutlich von jener der ICD-10 und auch von dem aktuellen DSM‑5. Die DSM-5-Überarbeitung sollte nur auf Basis empirischer Befunde erfolgen [12, 13] und führte zur Erweiterung der PTBS auf insgesamt 20 Symptome in 4 Clustern. Die Einführung der ICD-11 im deutschen Gesundheitssystem ist geplant, jedoch noch nicht terminiert und eine Überleitungstabelle der ICD-10- in ICD-11-Codes zur Erfassung von Mortalität und Morbidität wurde noch nicht publiziert [14].

Traumatische Erfahrungen sind mit einer Prävalenz zwischen 30 % und 56 % ein häufiger Belastungsfaktor im Kindes- und Jugendalter. Nach einem solchen Ereignis liegt die Punktprävalenz einer PTBS zwischen 4,2 % und 15,9 % [] [15,16], die Lebenszeitprävalenz bis zum Alter von 18 Jahren bei 7,8 % [17]. Eine unbehandelte PTBS kann zu chronifizierter Symptomatik und Langzeitfolgen bis ins Erwachsenenalter führen, wie zu weiteren psychischen Störungen, körperlichen Erkrankungen und Schlafstörungen [18, 19]. Im Vergleich zu Erwachsenen sind die Prävalenzraten für das Vollbild der PTBS bei Kindern und Jugendlichen geringer [20–23], wobei speziell im Kindes- und Jugendalter auch posttraumatische Stresssymptomatik unterhalb der diagnostischen Schwelle zu klinisch relevanter Belastung führt [24]. Die geringeren Prävalenzraten erklären sich allerdings nicht nur durch den Zusammenhang zwischen Lebensalter und Anzahl erlebter traumatischer Ereignisse. Die Ausprägung posttraumatischer Stresssymptome kann sich im jüngeren Alter von jenen im Erwachsenenalter unterscheiden ([24–28] ; Tab. 1).

**Table 1:** 

Wiedererleben	Weitere Symptome
*Jüngere Kinder und Vorschulalter*	
Reinszenierendes Spiel oder Verhalten Unspezifische Alpträume	Regressiver Verlust bereits erworbener Entwicklungsschritte, zeigt sich z. B. in Form von Mutismus (psychogenes Schweigen), Bettnässen, Nahrungsverweigerung, Schlafstörungen Zunahme alterstypischer Ängste, Trennungsängste, eingeschränktes Explorationsverhalten Insbesondere im Vorschulalter: Wutausbrüche, erhöhte Reizbarkeit, Enuresis (unwillkürlicher Harnabgang)/Enkopresis (willkürliches oder unwillkürliches Stuhlabsetzen an nicht dafür vorgesehenen Stellen)
*Schulalter*	
Repetitives Neu- oder Wiederinszenieren der Ereignisse im Spiel mit ergänzten Rettungsstrategien Unspezifische Alpträume	Konzentrations- und Aufmerksamkeitsprobleme in Verbindung mit schulischem Leistungsabfall Zunahme von Ängsten, erhöhtes Rückversicherungsverhalten Ein- und Durchschlafstörungen, unruhigerer Schlaf
*Jugendalter*	
Gedankliche Beschäftigung mit Entstellung oder Veränderung des eigenen Körpers	Verzögerung von Entwicklungsaufgaben (z. B. Autonomieentwicklung) Risikoverhalten, Substanzmissbrauch Selbstverletzendes Verhalten, Suizidalität

Zitiert aus Quellen [24–28]

Für eine entwicklungssensitive PTBS-Diagnostik wird empfohlen, altersangepasste Symptome beobachtbaren Verhaltens und eine reduzierte Anzahl obligatorischer Symptome heranzuziehen [24, 26]. Entwicklungsangepasste Formen der PTBS-Kriterien erwiesen sich als reliabel und valide zur Erfassung klinisch relevanter posttraumatischer Beeinträchtigungen bei Kindern und Jugendlichen [29, 30]. Diese alterssensiblen Vorschläge und ihre Evidenz wurden bislang in den Diagnosemanualen in Teilen berücksichtigt. Im DSM‑5 wurde eine Version der PTBS-Kriterien für Vorschulkinder bis zu einem Alter von 6 Jahren formuliert, jedoch sind die DSM-5-Kriterien auch für Jugendliche nicht uneingeschränkt geeignet, da das Symptomprofil der Betroffenen im Vergleich zu diesen komplexer ist [31]. In der englischen Ausgabe der ICD-11 werden entwicklungsabhängige Symptomvarianten formuliert, die aber in der deutschen Übersetzung noch fehlen. Die Möglichkeit einer reduzierten Symptomzahl wird in keinem Diagnosemanual explizit vorgeschlagen. Eine weitere diagnostische Herausforderung bei Kindern und Jugendlichen stellt mit Einführung der kPTBS-Diagnose die Beurteilung der SSO-Symptome dar. Ausprägungen in diesen Bereichen können auch entwicklungsbedingte Schwankungen der Selbstregulation abbilden, wie z. B. altersangemessene Wutausbrüche bei Kindern oder Stimmungsschwankungen sowie ein sich veränderndes Selbstbild bei Jugendlichen [25, 32].

Bisherige Befunde zur Anwendbarkeit der ICD-11-PTBS- und -kPTBS-Kriterien bei Kindern und Jugendlichen stützen die Konstruktvalidität beider Diagnosen [33, 34]. Die ICD-11-PTBS-Kriterien führen im Vergleich zu Versionen der Kriterien in DSM-IV, DSM‑5 und ICD-10 eher zu sinkenden [34–37] bis maximal ähnlich hohen Häufigkeitsraten [38, 39]. Drei Studien untersuchten, welche der ICD-11-Cluster bei Kindern und Jugendlichen für die PTBS- oder kPTBS-Diagnosestellung nicht hinreichend erfüllt waren. Dabei zeigten sich die geringsten Häufigkeitsraten bei dem Cluster „Übererregung“ bei Jugendlichen mit DSM-IV-PTBS nach physischem oder sexuellem Missbrauch [35], bei den Clustern „Wiedererleben“ und „Übererregung“ bei traumatisierten Kindern und Jugendlichen mit entwicklungsangepasster DSM-IV-Diagnose [36] sowie dem Cluster „Wiedererleben“ bei traumatisierten Kindern nach Naturkatastrophen [40]. Weitere Studien zeigten, dass sich die Beeinträchtigung der Kinder und Jugendlichen, welche die Diagnose nach ICD-11 erfüllten, nicht von denjenigen unterschied, die zwar andere Versionen der PTBS-Kriterien erfüllten, aber nach ICD-11 nicht mehr die Diagnose erhalten würden [35, 38, 41]. Dies könnte zu einer fehlenden Indikation für eine traumafokussierte Therapie bei einer relevanten Anzahl beeinträchtigter Kinder und Jugendlicher führen.

Dabei fanden entwicklungssensitive Anpassungen, die ab Januar 2023 in die ICD-11-PTBS-Kriterien aufgenommen wurden, noch keine Berücksichtigung in der Methodik der Studien oder in den verwendeten Instrumenten. Die vorliegende Arbeit adressiert 2 Forschungslücken in diesem Spannungsfeld: Zum einen wurden in den bisherigen Untersuchungen für den Vergleich von PTBS-Häufigkeitsraten anhand verschiedener Diagnosemanuale und auch für die Untersuchung der ICD-11-PTBS- und -SSO-Cluster bei Kindern und Jugendlichen weder für ICD-11 validierte Fragebögen eingesetzt noch aktuelle entwicklungsspezifische Vorschläge berücksichtigt. Zum anderen liegen noch keine empirischen Daten zur Einschätzung von Veränderungen im Bereich der SSO-Symptome bei Kindern und Jugendlichen als entwicklungsbedingt oder traumabedingt vor. Als erste Annäherung an eine solche diagnostische Einschätzung soll in dieser Studie die Attribution von SSO-Symptomen als ereignis- oder entwicklungsbedingt durch Bezugspersonen dienen. Die diagnostische Relevanz der Unterscheidung entwicklungsbedingt versus traumabedingt soll durch die Untersuchung des jeweiligen Zusammenhangs mit der PTBS-Symptomschwere der Kinder und Jugendlichen exploriert werden.

## Methode

### Studiendesign

Die vorliegenden Daten wurden im Rahmen der Validierung des „Child and Adolescent Trauma Screen-2“ (CATS-2) in deutschen psychosomatischen und psychiatrischen Kliniken erhoben. Eine detaillierte Beschreibung des Studienablaufs ist bei Sachser und Kollegen [42] dargestellt. Geschulte Projektmitarbeitende führten zwischen September 2019 und November 2020 eine querschnittliche Befragung mit traumatisierten Kindern und Jugendlichen und jeweils einer Bezugsperson durch. Alle Teilnehmenden waren Patient:innen in deutschen Versorgungseinrichtungen und erfüllten die Einschlusskriterien: Alter zwischen 7 und 17 Jahren, ausreichende Deutschkenntnisse, mindestens ein traumatisches Ereignis in der Lebensgeschichte (erhoben mit der CATS‑2 Checkliste). Die Ethikkommissionen der teilnehmenden Zentren (Ulm, Eichstätt) stimmten dem Vorgehen zu. Die Sorgeberechtigten wurden über das Vorgehen aufgeklärt und gaben ihr schriftliches Einverständnis.

### Teilnehmende

Insgesamt *N* = 88 deutsche Kinder und Jugendliche (7–17 Jahre, *M* = 14,15 Jahre, *SD* = 2,66, 68 % weiblich) und jeweils eine Bezugsperson nahmen an der CATS-2-Validierungsstudie teil. Von den Bezugspersonen füllten *N* = 79 die Fragebögen aus. Eine Beschreibung der demografischen Daten liefert Tab. 2.

**Table 2:** 

Charakteristika	Selbstbericht	Fremdbericht	Teststatistik
Alter, *M (SD)*	14,15 (2,66)	/	/
Geschlecht, *n* (% weiblich)	60 (68,2)	/	/
*Lebenssituation, n (%)*			
Biologische Eltern	75 (85,2)	/	/
Pflegeeltern/Adoptiveltern	3 (3,4)	/	/
Jugendhilfeeinrichtung	9 (10,2)	/	/
Andere	1 (1,1)	/	/
Anzahl traumatischer Ereignisse, *M (SD)*^*1*^	4,57 (2,04)	3,78 (2,19)	*t(62*) = 2,77**
*PTBS-Symptomschwere CATS‑2*			
DSM-5-PTBS	30,75 (12,91)	27,80 (11,91)	*t*(78) = 2,06*
ICD-11-PTBS	9,89 (4,59)	6,76 (4,39)	t(78) = 4,29**
ICD-11-SSO	8,90 (5,03)	8,67 (4,12)	*t*(78) = 0,47
ICD-11-kPTBS	18,78 (8,78)	15,43 (7,44)	*t*(78) = 2,74**

*CATS‑2* Child and Adolescent Trauma Screen‑2, *DSM‑5* 5. Auflage des „Diagnostic and Statistical Manual of Mental Disorders“, *ICD-11* 11. Auflage der „Internationalen statistischen Klassifikation der Krankheiten und verwandter Gesundheitsprobleme“, *kPTBS* komplexe posttraumatische Belastungsstörung, *M (SD)* Mittelwert (Standardabweichung), *PTBS* posttraumatische Belastungsstörung, *SSO* Schwierigkeiten in der Selbstorganisation und -regulation

*p < 0,05, **p ≤ 0,01

^1^Range Selbstbericht: 1–8, Fremdbericht: 0–8

### Operationalisierung

Mit dem 3‑teiligen Screeninginstrument *Child and Adolescent Trauma Screen 2* (CATS‑2; [42]) wird die posttraumatische Stresssymptomatik nach DSM‑5 und ICD-11 bei Kindern und Jugendlichen zwischen 7 und 17 Jahren erfasst. Im ersten Teil werden 15 potenziell traumatische Ereignisse anhand einer dichotomen Skala (*Ja/Nein*) abgefragt (Ereignis-Checkliste). Im zweiten Teil wird mit 25 Fragen auf einer 4‑stufigen Likert-Skala (0 = *nie* bis 3 = *fast immer*) die Häufigkeit der posttraumatischen Stresssymptome in den vergangenen 4 Wochen nach DSM-5-PTBS-, ICD-11-PTBS- und kPTBS-Kriterien erfasst und neben den allgemeinen Kriterien auch Symptomvarianten abgefragt, wie sie bei Kindern und Jugendlichen auftreten können (z. B. Wiedererleben auch in Form von Spiel). In die Auswertung der DSM-5-PTBS-Symptomschwere fließen 20 Items ein. Der Fragebogen erlaubt verschiedene dimensionale Auswertungen: DSM-5-PTBS-Symptomschwere (Range 0–60), ICD-11-PTBS-Symptomschwere (Range 0–18), ICD-11-kPTBS-Symptomschwere (Range 0–36) und Cut-off-Werte für Stresssymptomatik nach DSM-5-PTBS (≥ 21: erhöht, ≥ 25: hoch) sowie ICD-11-PTBS (≥ 7: erhöht, ≥ 9: hoch). Für die ICD-11-kPTBS liegen noch keine validierten Grenzwerte vor, es werden Summenwerte von ≥ 13 als positives Screening und ≥ 16 für eine wahrscheinliche kPTBS vorgeschlagen. Für eine kategoriale Auswertung des CATS‑2 schlagen die Autor:innen vor, Antworten von 2 (*oft*) oder 3 (*fast immer*) als klinisch relevante Ausprägung eines Symptoms anzusehen. Der dritte Teil des CATS‑2 erhebt mit 5 Fragen (Antwortformat: *Ja/Nein*) die Beeinträchtigung in wichtigen Funktionsbereichen. Der Fragebogen liegt in parallelen Versionen für ein Selbsturteil der Kinder und Jugendlichen und ein Fremdurteil der Bezugspersonen vor. Die interne Konsistenz für beide Versionen war in der vorliegenden Untersuchung sehr gut (Cronbachs Alpha Selbstbericht: α = 0,92; Fremdbericht: α = 0,91).

Der *Fragebogen zur Attribution der SSO-Kriterien* erhebt im Bezugspersonenbericht für jedes der 6 SSO-Symptome der ICD-11-kPTBS, ob bei Vorliegen des Symptoms beim Kind oder dem/der Jugendliche:n die Veränderung im jeweiligen Bereich auf ein traumatisches Ereignis oder den aktuellen Entwicklungsstand attribuiert wird. Eine Beispielfrage lautet: „Meinem Kind fällt es schwer sich zu beruhigen, wenn es aufgebracht (wütend, ängstlich, traurig) ist. Bitte geben Sie an, woran diese Veränderung Ihrer Meinung nach am meisten liegt.“ Die Antworten werden auf einer 5‑stufigen Skala erhoben (−2 = *wegen des Ereignisses*, −1 = *eher wegen des Ereignisses*, 0 = *trifft nicht zu*, 1 = *eher wegen des Alters*, 2 = *entspricht dem Alter*). Die Fragen wurden auf Grundlage der ICD-11-SSO-Kriterien [1, 4] formuliert und in einer Fokusgruppe mit Praktiker:innen und Forscher:innen aus dem Bereich der PTBS bei Kindern und Jugendlichen überarbeitet. Abschließend wurde die Verständlichkeit und Durchführbarkeit mit 2 Eltern von Betroffenen getestet. Die interne Konsistenz war akzeptabel (α = 0,74).

### Datenanalyse

Statistische Analysen wurden mithilfe des IBM Statistical Package for the Social Sciences SPSS 26.0 durchgeführt. Der Anteil fehlender Werte war sehr gering im CATS-2-Selbstbericht (0,2 %), im CATS-2-Fremdbericht (4,1 %) und im Fragebogen zur Attribution der kPTBS-Kriterien (3,0 %). Die Analyse fehlender Werte zeigte völlig zufälliges Fehlen („missing completely at random“), daher sollte die Analyse nur vollständiger Fälle keine verzerrten Schätzer geliefert haben [43, 44].

Unterschiede zwischen der Selbstauskunft der Kinder und Jugendlichen und der Fremdauskunft der Bezugspersonen wurden mit gepaarten t‑Tests analysiert. Anhand des kategorialen Auswertungsalgorithmus des CATS‑2 wurden wahrscheinliche PTBS-Diagnosen nach DSM‑5 und ICD-11 gestellt. Explorativ wurde mit den entsprechenden Items auch die PTBS-Diagnose nach ICD-10 gestellt. Zur Berücksichtigung der aktuellsten ICD-11-PTBS-Kriterien (Stand 01/2023) wurde ergänzend ein entwicklungsangepasster Algorithmus berechnet mit selbstschädigendem und -verletzendem Verhalten und Konzentrationsschwierigkeiten als weiteren Übererregungssymptomen und Beibehaltung der diagnostischen Schwelle von mind. 1 Symptom in diesem Cluster. Das Maß der Übereinstimmung von DSM-5-, ICD-10- und ICD-11-PTBS-Häufigkeiten wurde mit Cohens Kappa bestimmt. Im Hinblick auf ICD-11 wurden 2 Diagnosegruppen gebildet: keine ICD-11-PTBS sowie eine zusammengefasste Gruppe von ICD-11-PTBS und ICD-11-kPTBS. Für diese Gruppen wurden die Häufigkeitsraten der 6 ICD-11-kPTBS-Cluster berechnet und die mittlere Ausprägung der CATS-2-Symptomschwere mit einem t‑Test verglichen. Häufigkeitsraten jeder Skalenstufe der Attribution der SSO-Symptome wurden deskriptiv ausgewertet. Schließlich wurden für Fälle mit einem Fremdbericht anhand linearer Regressionen Zusammenhänge der Attribution von SSO-Symptomen als „ereignisbedingt“ oder „altersbedingt“ (Prädiktoren) mit der PTBS-Symptomschwere und SSO-Symptomschwere analysiert (Kriterien). Die Regressionsanalysen wurden jeweils für den Selbst- und Fremdbericht durchgeführt, außerdem wurde das Alter der Kinder und Jugendlichen zur Kontrolle eines Alterseffekts in das Modell aufgenommen. Dafür wurden 2 Attributionsvariablen gebildet: Der Betrag der Antworten „eher wegen des Ereignisses“ und „wegen des Ereignisses“ wurde zu einer Variable „ereignisbedingt“ aufsummiert. Analog wurden die Antworten „eher wegen des Alters“ und „altersbedingt“ zur Variable „altersbedingt“ aufsummiert. Da die Analysen explorativ waren, wurde die Vorwärts-Selektion der Prädiktoren erlaubt [45].

## Ergebnisse

### Stichprobencharakteristika

Deskriptive Charakteristika der Studienteilnehmenden sind in Tab. 2 dargestellt. Im Mittel lagen die CATS-2-Summenwerte für DSM-5-PTBS und ICD-11-kPTBS sowohl im Selbst- als auch im Fremdbericht über den Cut-off-Werten für eine erhöhte posttraumatische Stresssymptomatik (≥ 21 für DSM-5-PTBS bzw. ≥ 13 für ICD-11-kPTBS). Der ICD-11-PTBS-Summenwert lag für den Selbstbericht im auffälligen Bereich (≥ 7), jedoch nicht für den Fremdbericht. Im Selbstbericht gaben teilnehmende Kinder und Jugendliche im Vergleich zu ihren Bezugspersonen eine höhere Anzahl traumatischer Ereignisse und eine höhere Symptomschwere der DSM-5-PTBS, ICD-11-PTBS und ICD-11-kPTBS an.

### Häufigkeitsraten der ICD-11-PTBS- und kPTBS-Symptomcluster

Die kategoriale Diagnoseschätzung ergab im Vergleich verschiedener PTBS-Kriterien geringe Unterschiede zwischen DSM‑5 und ICD-10, sowohl im Selbstbericht (DSM-5-PTBS *n* = 63, 71,6 %; ICD-10-PTBS *n* = 63, 71,6 %; ICD-11-PTBS *n* = 10, 11,4 %; ICD-11-kPTBS *n* = 20, 22,7 %,) als auch im Fremdbericht (DSM-5-PTBS *n* = 38, 48,1 %; ICD-10-PTBS *n* = 34, 43,0 %; ICD-11-PTBS *n* = 2, 2,9 %; ICD-11-kPTBS *n* = 13, 18,8 %). Bei Anwendung der ICD-11-Kriterien zeigten sich im Vergleich zu DSM‑5 und ICD-10 geringere Häufigkeitsraten jeweils für PTBS und auch kPTBS. Auch für PTBS und kPTBS zusammengenommen unterschieden sich die Häufigkeitsraten von ICD-11 um mindestens 20 % von DSM‑5 und ICD-10. Mit dem entwicklungsangepassten Algorithmus für ICD-11, unter Berücksichtigung weiterer entwicklungssensitiver Symptome und Beibehaltung der diagnostischen Schwelle von einem Symptom pro Cluster, stiegen die Häufigkeitsraten für PTBS im Selbstbericht um 6,8 Prozentpunkte, für kPTBS um 4,6 Prozentpunkte an. Im Fremdbericht stiegen die Häufigkeitsraten für PTBS um 3,4 Prozentpunkte und für kPTBS um 0,7 Prozentpunkte an (Selbstbericht: ICD-11-PTBS *n* = 16, 18,2 %; ICD-11-kPTBS *n* = 24, 27,3 %; Fremdbericht: ICD-11-PTBS *n* = 5, 6,3 %; ICD-11-kPTBS *n* = 15, 19,0 %). Insgesamt blieben die Häufigkeitsraten auch für den entwicklungsangepassten ICD-11-Algorithmus geringer als für ICD-10 und DSM‑5. Die diagnostische Übereinstimmung zwischen der zusammengefassten Gruppe von ICD-11-PTBS und -kPTBS mit DSM‑5 und ICD-10-PTBS war im Selbstbericht schwach (beide *κ* = 0,34), im Fremdbericht mäßig (DSM‑5 *κ* = 0,47, ICD-10 *κ* = 0,49), für den entwicklungsangepassten Algorithmus im Selbstbericht mäßig (beide *κ* = 0,50) und im Fremdbericht gut (*κ* = 0,64). Die diagnostische Übereinstimmung zwischen DSM-5- und ICD-10-PTBS war in beiden Berichten sehr gut (beide *κ* > 0,80). Teilnehmende mit ICD-11-(k)PTBS oder der Diagnose nach dem angepassten ICD-11-PTBS-Algorithmus stellten dabei eine Teilgruppe derjenigen dar, die PTBS nach ICD-10 oder DSM‑5 erfüllten.

**Figure 1:**
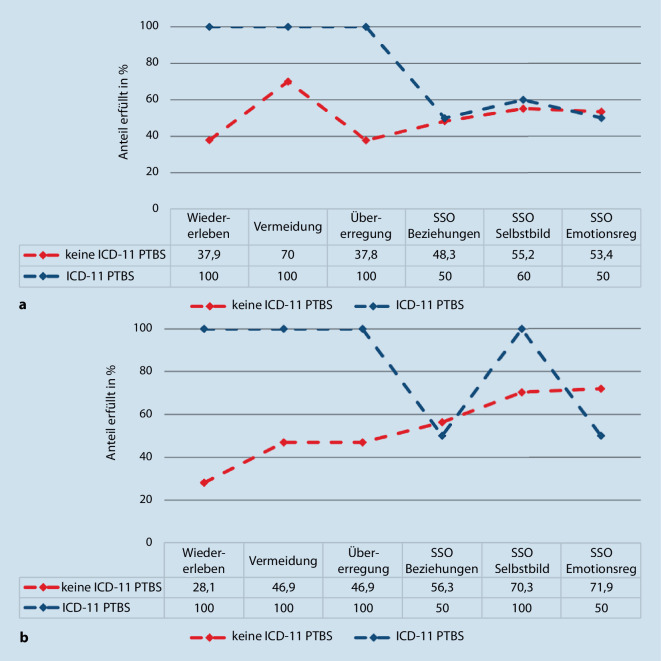

Hinsichtlich der Häufigkeiten von Personen in der vorliegenden Stichprobe, die Symptome oberhalb der diagnostischen Schwelle für ICD-11 in den (k)PTBS-Clustern angaben, zeigten sich im Vergleich der Symptomcluster die geringsten Raten für die Gruppe ohne ICD-11-Diagnose im Selbstbericht in den Clustern „Wiedererleben“ (37,9 %) und „Übererregung“ (37,8 %), im Fremdbericht im Cluster „Wiedererleben“ (28,1 %; Abb. 1a,b). Bei Anwendung des entwicklungsangepassten Algorithmus zeigte sich die geringste Häufigkeit im Cluster „Wiedererleben“ (Selbstbericht: 25,0 %, Fremdbericht: 19,1 %). Die zusammengefasste Gruppe von ICD-11-PTBS und -kPTBS berichtete im Mittel höhere PTBS-Symptomschwere (*M* = 40,67; *SD* = 7,83) als die Gruppe ohne ICD-11-PTBS (*M* = 25,62, *SD* = 12,03, *t*(86) = 6,20, *p* < 0,001). Beide Gruppen liegen im Durchschnitt oberhalb des Cut-offs für klinisch relevante Symptomatik. Dieser Effekt blieb auch im Vergleich mit dem entwicklungsangepassten ICD-11-PTBS-Algorithmus signifikant und die Mittelwerte lagen über dem Cut-off (ICD-11-PTBS und -kPTBS: *M* = 39,88, *SD* = 7,86; keine ICD-11-PTBS: *M* = 23,15, *SD* = 11,29, *t(*86) = 7,91, *p* < 0,001).

### Attribution der SSO-Symptome aus Bezugspersonensicht

Tab. 3 fasst die Anzahl der Antworten im Fragebogen zur Attribution der SSO-Kriterien zusammen. Am häufigsten wurden Veränderungen im SSO-Symptombereich auf ein traumatisches Ereignis attribuiert (52,6 %). Ein Viertel (25,2 %) aller Antworten gab keine Symptomatik an und insgesamt 22,2 % der Einschätzungen attribuierten Veränderungen auf das Alter der Betroffenen. Die Ergebnisse der Regressionsanalysen sind in Tab. 4 dargestellt. Ein höheres Alter zeigte einen signifikanten Zusammenhang mit PTBS-Symptomschwere im Selbst- und Fremdbericht sowie mit SSO-Symptomschwere im Selbstbericht. Die ICD-11-PTBS-Symptomschwere im Fremdbericht hing signifikant mit der Einschätzung der SSO-Veränderungen als ereignisbedingt zusammen, nicht jedoch mit der Attribution altersbedingt. Die ICD-11-SSO-Symptomschwere hing mit der Einschätzung als ereignisbedingt und auch als altersbedingt zusammen. Die ICD-11-PTBS- und -SSO-Symptomschwere im Selbstbericht zeigte keinen Zusammenhang mit der angegebenen Attribution der SSO-Symptome und die Modelle der Selbsteinschätzung hatten eine geringere Varianzaufklärung.

**Table 3:** 

SSO-Kriterien, *n* (%)	Entspricht dem Alter	Eher wegen des Alters	Trifft nicht zu	Eher wegen des Ereignisses	Wegen des Ereignisses
Reizbarkeit	9 (12,0)	16 (21,3)	12 (16,0)	19 (25,3)	16 (21,3)
Verminderte Emotionalität	3 (4,0)	11 (14,7)	19 (25,3)	26 (34,7)	16 (21,3)
Schuld	2 (2,7)	10 (13,3)	17 (22,7)	21 (28,0)	24 (32,0)
Wertlosigkeit	1 (1,3)	16 (21,3)	12 (16,0)	25 (33,3)	19 (25,3)
Emotionale Distanz	3 (4,0)	11 (14,7)	26 (34,7)	27 (36,0)	7 (9,3)
Beziehungsgestaltung	4 (5,3)	10 (13,3)	26 (34,7)	22 (29,3)	12 (16,0)
Summe	22 (4,9)	77 (17,3)	112 (25,2)	140 (31,5)	94 (21,1)

*N* = 75 ausgefüllte Fragebögen

**Table 4:** 

			95 % Konfidenzintervall		
Effekte	*b*	*SE*	Unterer Wert	Oberer Wert	*p*
*ICD-11-PTBS-Symptomschwere (Selbstbericht)*^*a*^					
Alter	0,66	0,20	0,26	1,06	0,003
*ICD-11-SSO-Symptomschwere (Selbstbericht)*^*b*^					
Alter	0,65	0,22	0,20	1,09	0,005
*ICD-11-PTBS-Symptomschwere (Fremdbericht)*^*c*^					
Ereignisbedingt	0,72	0,57	0,41	1,03	< 0,001
Alter	0,55	0,18	0,00	0,20	0,003
*ICD-11-SSO-Symptomschwere (Fremdbericht)*^*d*^					
Ereignisbedingt	1,07	0,15	0,78	1,37	< 0,001
Altersbedingt	1,31	0,32	0,67	1,94	< 0,001

Nur signifikante Prädiktoren wurden dargestellt; Einschluss: Vorwärts-Selektion

^a^Modell: F(2, 68) = 10,82, *p* = 0,002, korrigiertes *R*^*2*^ = 0,12

^b^Modell: F(1, 69) = 8,49, *p* = 0,005, korrigiertes *R*^*2*^ = 0,10

^c^Modell: *F*(2, 68) = 17,10 *p* < 0,001, korrigiertes *R*^*2*^ = 0,31

^d^Modell: *F*(2, 68) = 26,26 *p* < 0,001, korrigiertes *R*^*2*^ = 0,42

## Diskussion

Die vorliegende Studie untersuchte die entwicklungsbezogene Passung der ICD-11-PTBS- und kPTBS-Kriterien, erfasst mit dem CATS-2-Fragebogen, in einer Inanspruchnahme-Stichprobe deutscher Kinder und Jugendlicher, die mindestens ein traumatisches Ereignis erlebt haben. Die Teilnehmenden berichteten im Mittel mehrere traumatische Ereignisse und zeigten für DSM‑5 im Selbst- und Fremdbericht sowie für ICD-11 im Selbstbericht posttraumatische Belastungssymptome oberhalb der klinisch relevanten Cut-off-Werte. In dieser belasteten Stichprobe zeigten sich geringere Angaben im Fremdbericht in Bezug auf die Anzahl der traumatischen Ereignisse (5 versus 4) und die Ausprägung der PTBS-Symptomatik verglichen mit dem Selbstbericht. Dieser Unterschied ist konsistent mit bisherigen Erhebungen bei jungen Patient:innen [46] und betont die Wichtigkeit der selbstberichteten Symptome. Bezugspersonen unterschätzen durchgehend die Belastung der betroffenen Kinder und Jugendlichen.

Die Kriterien der ICD-11-PTBS haben in der vorliegenden Untersuchung zu geringeren Häufigkeitsraten geführt als die ICD-10- und DSM-5-PTBS. Dabei fällt der Unterschied zwischen Selbst- und Fremdbericht für die ICD-11-PTBS größer aus im Vergleich zu DSM-5-PTBS oder ICD-11-kPTBS. Der CATS-2-Fragebogen ermöglicht dabei durch die parallele Konzeption für DSM‑5 und ICD-11 bereits die Berücksichtigung einiger der vorgeschlagenen entwicklungssensitiven Anpassungen der Kriterien. Werden entwicklungsangepasste PTBS-Symptome zusätzlich zur Diagnoseschätzung hinzugezogen, so identifiziert der in der vorliegenden Arbeit angewandte ICD-11-Algorithmus deutlich mehr (k)PTBS-Fälle. Im Vergleich zu DSM‑5 und ICD-10 bleiben die Häufigkeitsraten dabei weiterhin geringer. Die geringeren Häufigkeitsraten bei Anwendung der ICD-11-PTBS-Kriterien im Kindes- und Jugendalter werden durch Vorbefunde gestützt [25, 27, 32]. Die Ergebnisse dieser Studie deuten an, dass die entwicklungssensitiven Symptome eine passende Ergänzung für junge Betroffene sind und sollten bei weiteren Anpassungen von Fragebögen berücksichtig werden.

Sinkende Häufigkeitsraten für PTBS bei traumatisierten Kindern und Jugendlichen im Vergleich zu ICD-10 und DSM‑5 könnten als Folge des ICD-11-Reformziels, eine geringere Überschneidung mit Kriterien anderer Störungen zu erreichen [6, 47], bewertet werden und diese Ziele erfüllen. Eine Reduktion auf Kernsymptome ermöglicht eine einfachere Diagnostik, differentialdiagnostische Abklärung und passgenaue Interventionsplanung, geringere Komorbiditätsraten können Behandlungsentscheidungen erleichtern.

Im Falle dennoch weiterhin vorliegender Komorbidität empfiehlt die deutsche Leitlinie eine parallele oder sequentielle Behandlungsplanung [48]. Für Kinder und Jugendliche sind im Weiteren prospektive Studien notwendig, um beurteilen zu können, ob die Behandlung anderer vorliegender Diagnosen insgesamt zu einer ausreichenden Reduktion der Belastung und Funktionseinschränkungen führt und die nach ICD-11 dann subklinische PTBS im Verlauf ausreichend abklingt.

Demgegenüber steht die Argumentation einzelner Publikationen zu PTBS im Kindes- und Jugendalter, die bereits vor der Neufassung der Diagnosemanuale DSM‑5 und ICD-11 andere weiter gefasste Versionen der PTBS-Kriterien für diesen Altersbereich als zu restriktiv kritisiert haben hinsichtlich der Abbildung beeinträchtigender posttraumatischer Stresssymptomatik [27, 49]. Die ICD-11-Kriterien scheinen noch enger gefasst und identifizieren auch unter Berücksichtigung entwicklungssensitiver Symptome weniger Fälle. Auch Teilnehmende, die keine ICD-11-PTBS oder -kPTBS erfüllten, gaben im Mittel eine klinisch relevante posttraumatische Symptombelastung an, sowohl für den diagnostischen Algorithmus nach CATS‑2 als auch für die entwicklungsangepasste Version. Damit könnten die Daten darauf hinweisen, dass mit Anwendung der ICD-11-Kriterien die Behandlungsindikation für eine traumafokussierte Psychotherapie für einen Teil junger Personen entfallen wird, der unter einer ausgeprägten posttraumatischen Stresssymptomatik leidet.

Diejenigen Teilnehmenden, die eine ICD-11-PTBS- oder -kPTBS-Diagnose verfehlen, erfüllen diese aufgrund geringerer Wiedererlebenssymptome im Selbst- oder Fremdbericht nicht. Dieser Befund bestätigt die bisherige Studienlage, in der die geringsten Häufigkeitsraten für „Übererregung“ oder „Wiedererleben“ berichtet wurden [35, 36, 40] anhand eines für ICD-11-PTBS und -kPTBS validierten Fragebogens. Ein möglicher Grund hierfür könnte in der Auswahl der ICD-11-Symptome in diesem Symptomcluster liegen. Das Wiedererleben traumatischer Erinnerungen wird im CATS-2-Fragebogen durch Flashbacks und Alpträume erfasst. So wurden die Wiedererlebenssymptome in frühen Vorstellungen der ICD-11-PTBS-Kriterien beschrieben, als Auswahl der Wiedererlebenssymptome, die PTBS-spezifisch sind, ohne Überschneidung mit Kriterien anderer Störungen [23], und auch in anderen ICD-11-basierten PTBS-Fragebögen übernommen [46, 50]. Durch die Berücksichtigung entwicklungsspezifischer Symptomvarianten in den ICD-11-Kriterien wird ein breiteres Spektrum an Übererregungssymptomen erfasst. Diese Spezifizierungen sollten, in der jeweils aktuellen Fassung der ICD-11-(k)PTBS-Kriterien, in künftigen Aktualisierungen des CATS-2-Fragebogens und auch anderer ICD-11-PTBS-Screening-Fragebögen Berücksichtigung finden.

SSO-Symptome werden aus Sicht der Bezugspersonen überwiegend den traumatischen Ereignissen zugeschrieben. Ein statistischer Zusammenhang der SSO-Symptomzuschreibungen mit der Symptomschwere zeigt sich nur für den Fremdbericht, nicht jedoch für den Selbstbericht: Sowohl die ICD-11-PTBS als auch die SSO-Symptomatik werden als schwerwiegender eingeschätzt, je ausgeprägter die Zuschreibung der SSO-Symptome an das traumatische Ereignis ist. Dies stützt die Konstruktvalidität des Fremdberichts. Die Schwere der SSO-Symptomatik hängt zudem auch mit einer Attribution als altersbedingt zusammen, auch nach Kontrolle des Einflusses des Alters. Durch die Zusammenhänge wird ein hoher Anteil der Varianz der SSO-Symptomschwere im Fremdbericht aufgeklärt. Diese Studie liefert erstmals Ergebnisse zur Attribution der SSO-Symptome und zeigt, dass Bezugspersonen zwischen altersbedingter und ereignisbedingter Ausprägung dieser Symptome unterscheiden. Fehlende Zusammenhänge der Attribution der SSO-Symptome aus Bezugspersonensicht mit dem Selbstbericht der Betroffenen können in der durchgehenden Divergenz zwischen Bezugspersonen- und Betroffenensicht (z. B. [33, 37]) begründet sein, weiterhin könnten Kinder und Jugendliche eventuell Gründe für Veränderungen im SSO-Bereich selbst besser oder auch schlechter einschätzen. Bezüglich der SSO-Symptome erhebt der CATS-2-Fragebogen die Häufigkeit der DSM-5- und ICD-11-(k)PTBS-Symptome, ohne spezifischer den Zusammenhang der Veränderungen mit einem traumatischen Ereignis zu erfragen. Dies könnte insbesondere für die Einschätzung der SSO-Symptome ergänzt werden.

### Limitationen

Während diese Studie einen Beitrag zur Frage der entwicklungsspezifischen Passung der ICD-11-PTBS- und -kPTBS-Symptome liefert und erstmals einen für diese Kriterien validierten Fragebogen verwendete, müssen die Ergebnisse vor dem Hintergrund einiger Limitationen bewertet werden. Einerseits handelte es sich um eine querschnittliche Befragung in einer Inanspruchnahme-Stichprobe traumatisierter Kinder und Jugendlicher, wodurch keine Veränderung von Symptomen und Entwicklung über eine Zeitspanne abgebildet wird, welche gerade für die Beurteilung der SSO-Symptome als eher alters- oder entwicklungsbedingt hilfreich sein kann. Die Befragung von Bezugspersonen mit dem Fragebogen zur Attribution der SSO-Kriterien war rein explorativ. Der Fragebogen ist nicht validiert und die Ergebnisse können nur deskriptiv interpretiert werden. Die geschätzten Diagnosegruppen in der beschriebenen Auswertung beruhen nur auf Grenzwerten der Selbst- und Fremdauskunftsfragebögen. Diese Grenzwerte wurden zwar mit einem klinischen Interview validiert, eine sichere Diagnosestellung ist daraus jedoch beim Einsatz als rein selbstausgefülltes Instrument nicht ableitbar, wäre jedoch in einer interviewgestützten Nutzung der Fragebögen möglich. Darüber hinaus berücksichtigt der CATS-2-Fragebogen nicht die neuesten Aktualisierungen der ICD-11-(k)PTBS-Kriterien mit allen entwicklungsangepassten Empfehlungen.

### Implikationen

Die traumabezogene Abgrenzung der Veränderungen im SSO-Bereich von entwicklungsbedingten Schwankungen im Kindes- und Jugendalter stellt eine Herausforderung dar. Selbst- und Fremdbericht unterschieden sich diesbezüglich, daher ist es für eine valide Diagnosestellung notwendig, die Symptombelastung auch im Selbstbericht zu erfassen. Weitere Forschung zur Einschätzung der SSO-Symptome aus Sicht der Betroffenen selbst und auch im klinischen Urteil durch Fachpersonal sind als nächste Schritte notwendig. Ebenso sollten längsschnittliche Symptombeobachtungen für eine bessere differentialdiagnostische Abgrenzung von Psychopathologie und der Einordnung altersangemessener Schwankungen in diesen Bereichen durchgeführt werden.

Die Cut-off-Werte des CATS‑2 wurden anhand der Selbstberichtsversion des Fragebogens und eines klinischen Interviews mit den Betroffenen validiert [33] und gleichermaßen für den Selbstbericht als auch den Fremdbericht empfohlen. Die Durchführung beider Versionen im Kinder- und Jugendbereich ist aufgrund der beobachteten Diskrepanzen in Selbst- und Fremdbericht empfehlenswert. Im Fremdbericht sollten auch Summenwerte nahe des unteren Cut-offs als Indikation für ein klinisches Interview zur (k)PTBS interpretiert werden.

Es zeigte sich zudem, dass die für Erwachsene formulierten ICD-11-PTBS-Kriterien bei Kindern und Jugendlichen möglicherweise nur eingeschränkt klinisch relevante posttraumatische Belastung abbilden und die entwicklungsspezifischen Ergänzungen der ICD-11 in weitere Revisionen von Fragebögen übernommen werden sollten. Anschließend sind weitere Untersuchungen notwendig, um abzuschätzen, ob die in der ICD-11 vorgeschlagenen entwicklungssensitiven Symptomvarianten die Belastung ausreichend erfassen. Screening-Instrumente sollten ebenso in Validierungsstudien mit Anwendung der ICD-11-Textversion der Diagnosekriterien verglichen werden. Gleichzeitig ermöglicht der Einsatz von Screening-Fragebögen wie dem CATS‑2, die sowohl die ICD-11-PTBS-Symptomschwere als auch die DSM-5-PTBS-Symptomschwere erheben, die Abwägung der Behandlungsindikation im Einzelfall durch den Vergleich verschiedener Schwellenwerte.
